# Neuromodulatory Support for Breathing and Cardiovascular Action During Development

**DOI:** 10.3389/fped.2021.753215

**Published:** 2021-09-30

**Authors:** Ronald M. Harper, Kalpashri Kesavan

**Affiliations:** ^1^Department of Neurobiology and the Brain Research Institute, David Geffen School of Medicine, University of California, Los Angeles, Los Angeles, CA, United States; ^2^Department of Pediatrics, David Geffen School of Medicine, University of California, Los Angeles, Los Angeles, CA, United States

**Keywords:** neuromodulation, periodic breathing, apnea, cardiovascular, proprioception, prematurity

## Abstract

Neonatal survival requires precise control of breathing and cardiovascular action, with fatal consequences or severe injury without support. Prematurity presents multiple opportunities to disrupt cardiorespiratory regulation, leading to expressions of apnea of prematurity, periodic breathing, and inappropriate cardiovascular responses to apnea. Failed breathing control can result from altered breathing drives, typically arising from untimely development of sensory or motor coordination processes. Some drives, such as temperature, are a special concern in neonates with low body mass, enhancing susceptibility to rapid body cooling. Chemical drives, such as pH or CO_2_ or O_2_, may be inadequately developed; in some conditions, such as congenital central hypoventilation syndrome (CCHS), breathing responses to CO_2_ or low O_2_ may be reduced or absent, and coupling of cardiovascular responses to breathing changes are abolished. Sleep states exert profound influences on both chemical and temperature drives, with rapid eye movement (REM) sleep potentially modifying descending temperature influences, and state transitions significantly altering respiratory responses to chemical stimuli. In addition, neonates spend the majority of time in REM sleep, a state which induces a generalized inhibition of skeletal muscle activity that abolishes muscle tone to upper airway and thoracic wall muscles, enhancing the likelihood for obstructive sleep apnea. Although disrupted regulatory drives can often be replaced by positive (or negative) pressure ventilation, such as continuous positive airway pressure or enhanced by manipulating neurotransmitter action *via* caffeine, those approaches may exert negative consequences in the long term; the lungs of neonates, especially premature infants, are fragile, and easily injured by positive pressure. The consequences of caffeine use, acting directly on neural receptors, although seemingly innocuous in the near-term, may have long-term concerns and disrupts the integrity of sleep. The developmental breathing field needs improved means to support ventilation when one or more drives to respiration fail, and when the cardiovascular system, depending heavily on interactions with breathing, is compromised. Neuromodulatory procedures which manipulate the vestibular system to stabilize breathing or use tactile or proprioceptive stimuli to activate long-established reflexive mechanisms coupling limb movement with respiratory efforts can provide support for central and obstructive apnea, as well as for periodic breathing and cardiovascular action, particularly during sleep.

## Introduction

The rate of breathing and extent of air exchange in infants is normally controlled by sensing CO_2_ and O_2_, by core body temperature, and by voluntary breathing efforts, all of which are partially modulated by sensory feedback of lung and muscle stretch as well as by afferent signals from airflow. However, some respiratory drives, such as those from CO_2_ or O_2_ sensing, can be diminished, lost, or shifted in timing by altered development, or by genetic malformations, as in congenital central hypoventilation syndrome CCHS (1), or by neural trauma in the neonatal period (2). CCHS subjects show loss of CO_2_ and O_2_ sensing (3), as well as impaired temperature control on breathing. Other disease processes, such as Covid-19, can be accompanied by loss of the perception of dyspnea, inappropriate responses to CO_2_ or low O_2_, resulting in “tolerant tachypnea” and “happy hypoxemia” (4, 5).

Some instances of impaired breathing, particularly during sleep, result from disrupted cerebellar interactions with other regulatory structures, particularly the parabrachial pons. Stroke, especially with cerebellar injury, or other developmental injury with cerebellar involvement, e.g., Joubert's syndrome, is frequently accompanied by airway obstruction (2, 6), an outcome often a consequence of a loss of coordination, a principal cerebellar function, between reduced drives to upper airway muscles but continued diaphragmatic action, i.e., flaccid oropharyngeal muscles in the presence of negative thoracic pressures from a descending diaphragm. The loss of motor influences to upper airway muscles during the paralysis of REM sleep can thus result in obstructive sleep apnea (OSA). The parabrachial pons exerts a pivotal role in these sequences, coordinating signals from the cerebellum, vagal and thoracic afferents, integrating temperature and other input from more-rostral brain influences, while providing respiratory phase switching and state arousal actions (7). The state arousal functions are critical in recovery from some forms of apnea in developing infants.

The impact of sleep on reduced or distorted breathing drives extends to state influences on chemical (8), or temperature influences on breathing in early development, and can contribute to apnea of prematurity. Distortion in timing of respiratory drive influences, such as altered timing between central and peripheral chemoreceptor integration, can lead to periodic breathing. Some drives supporting breathing are lost during sleep, including voluntary breathing efforts, and the so-called “wakefulness stimulus to breathe” (9). Temperature influences on breathing, an important consideration in infants with low body mass and thus susceptible to low or high environmental temperatures, are a concern. Temperature drives are diminished during REM sleep in young or adult feline preparations (10, 11). Those REM-related effects apparently differ in premature human infants (12).

Premature infants are at high risk for cerebellar injury (hemorrhages, infarctions, and intermittent hypoxic exposure), which can compromise cerebellar growth and function (13, 14). Such cerebellar injury, seizures, or hypoxic damage can alter respiratory motor timing circuitry, or delay circulation, leading to a mismatch of central and peripheral chemoreception and modify timing of inspiratory signaling to respiratory muscles, all of which are heavily dependent on the integrity of deep cerebellar nuclei and control influences on those nuclei (15). These functional distortions can result in periodic breathing, a start-stop respiratory patterning leading to intermittent hypoxia exposure that is injurious to brain tissue (16). Such disturbed breathing patterns are common in premature infants, and can appear in full-term neonates. Severe inflammatory processes and white matter injury can occur as well (17). Appropriate and sustained inspiratory and expiratory timing must be restored to minimize periodic breathing, together with reclamation or replacements for breathing drives; that timing is heavily dependent on intact cerebellar and parabrachial pontine processes (7).

## Hyperpnea With Exercise, and a Phylogenetically-Old Reflexive Breathing Drive

The potential contributors to enhanced breathing efforts with exercise has been a target of respiratory physiologists for over a century. Increased ventilation to accommodate the additional metabolic demands is one possibility, but timing of the enhanced efforts before metabolic demands have built up argue against that interpretation. The range of potential mechanisms has been reviewed in detail (18), and that review described multiple processes that may be operating, with no single process providing a unifying, definitive mechanism.

A loss of drive from chemoreceptor sources, i.e., high CO_2_ or low pH, or from low O_2_, or insufficient temperature influences or loss of the “waking stimulus” during sleep can be overcome by recruiting a phylogenetically old reflex which couples limb locomotion with increased breathing-muscle activity. The coordination between limb movement and breathing can be easily observed in animals; a popular respiratory tale is that the distinguished French physiologist, DeJours, who loved horse racing, even in cold weather, observed synchrony between the visible expired air of horses with forward movement of the hoofs during running. Such coupling of ventilation to exercise has repeatedly been demonstrated (19, 20). In humans, children with CCHS, who express a mutation in PHOX2B leading to loss of sensitivity to CO_2_ and O_2_ (3), as well as poor temperature control (21), provide an “Experiment of Nature” which illustrates the role of movement to enhance breathing. Left to sit passively, as in watching television or playing a video game, CCHS patients fail to breathe and will rapidly lose oxygenation until provoked to do so by their caretaker to voluntarily breathe (voluntary breathing drives remain intact in CCHS children). However, if such children actively move about, as in playing soccer, they ventilate adequately (22). The limb movement can be passive; simple back and forth movement of the foot will sustain breathing, and does so, even during sleep (20, 23).

The coupling of movement with breathing provides species-survival benefits. The reflex immediately augments breathing in response to locomotion when the organism is threatened, and time is not available to enhance CO_2_ or other chemical processes usually used to increase ventilation. The independence from CO_2_ stimulation is an important aspect in premature infants with apnea of prematurity, because ventilatory responses to increasing CO_2_ may be poorly developed, secondary to diminished central sensitivity to CO_2_.

The action of both foot and limb movements can enhance breathing efforts; reflexive coupling of breathing with limb movements developed early phylogenetically, and is present in all four limbs used for locomotion. Thus, in humans, movement of both the upper and lower limbs can participate in enhancing ventilation, an aspect of importance in intervening for hypoventilation in spinal cord injured patients, where proprioceptive signaling from lower limbs may be lost (24). Proprioceptive signals from muscles and tendons of the hand and feet travel *via* peripheral neural pathways to the spinal cord, and then travel up the cord *via* spinocerebellar pathways to the brain stem and cerebellum (25).

Although selected neurons in the brainstem have respiratory pacemaker qualities to influence breathing rate, the timing and extent of those breathing signals depend heavily on multiple inputs, including descending forebrain signals, which include temperature influences from the hypothalamus and affective signals from the amygdala (26), as well as afferent signals from the lung, vascular system, and thoracic wall (18). Timing and extent of those signals are coordinated by cerebellar and pontine processes which integrate signaling to nuclei mediating breathing in the medulla and pons (20, 25, 27–29). Nuclei within the medulla, in turn, send signals to the phrenic motor pools C3, C4, and C5 of the spinal cord that innervate the diaphragm, cervical and thoracic spinal nerves serving ancillary respiratory intercostal muscles, and to the hypoglossal nuclei supplying the genioglossal fibers, the protruder (dilating) muscles of the tongue (30). The processes which maintain regularity in breathing, as well as momentary processes in respiratory effort depend on multiple inputs from forebrain and brainstem sites, as well as peripheral thoracic and oral airway sensory input. The different inputs require synchronization between inputs, and timely integration to maintain sufficient drive to maintain breathing, cessation of efforts when appropriate, and appropriate interactions with cardiovascular action to maintain appropriate perfusion.

## Failing Mechanisms in Sleep-Disordered Breathing; Failing Breathing “Drives” and Disrupted Timing

Several major issues confront breathing control in neonatal sleep; one is to prevent obstructive sleep apnea (OSA). A set of oro-pharyngeal muscles participate in OSA, with the tongue genioglossal muscles being principal dilators maintaining upper airway patency, and thus, a key target for OSA intervention (30). For neonatal breathing, maintaining airway patency is key to preventing OSA, but such maintenance is a concern with the generalized somatic muscle paralysis of REM sleep. The REM paralysis leads to flaccid upper airway musculature; however, diaphragmatic movements are maintained in REM sleep, producing high negative airway pressure, with the danger of collapsing the upper airway musculature, resulting in airway occlusion. Prevention of upper airway occlusion during REM sleep is thus dependent on maintaining upper airway muscle tone as well as supplying appropriate phasic *timing* of that tone to the muscles, i.e., dilating the airway slightly ahead of diaphragmatic descent and its resulting negative pressure.

A second concern in newborn breathing is protection against loss of temperature influences during the REM sleep reduction of descending hypothalamic influences. Respiratory rate is partially dependent on core temperature, which provides a significant drive to breathing in an infant with low body mass, making it susceptible to rapid core cooling when unclothed or placed in other low temperature conditions. These issues have been largely addressed in neonatal intensive care units with use of servo-controlled incubators as well as heated and humidified airflow to maintain normothermia; however, in less-developed or less-supportive circumstances, temperature control remains an issue.

A third concern is the alteration in impact of chemical sensing that accompanies state changes (8). In premature infants with apnea of prematurity, ventilatory responses to increasing CO_2_ are immature, secondary to diminished central sensitivity to CO_2_ (31–33). In some conditions, such as CCHS, chemical sensing of CO_2_ is sufficiently reduced that affected children need to be mechanically ventilated during sleep. Non-CCHS children also undergo less extreme state-related breathing responses to CO_2_ sensing, but the potential for impaired sensitivity with very early development remains a concern. Timing of inspiratory and expiratory efforts involves a complex interaction of integrating descending signals from rostral brain areas, such as affective regions of the amygdala and temperature influences from the hypothalamus, and afferent signals from stretch receptors of the lung. The cerebellum and parabrachial pons exert a coordination role in integration of these multiple inputs, especially through amygdala projections to the parabrachial pons, descending hypothalamic influences, and lung afferent projections.

Periodic breathing patterns result in successive exposure to intermittent hypoxia with each stopped-breath episode; in infants, the resulting hypoxia leads to desaturations to very low levels, with rapid restoration to normoxia with onset of the next burst of respiratory efforts. Although periodic breathing is sometimes ignored in neonates, the consequences to the brain and other structures can be severe. The repeated desaturation/reoxygenation sequence is injurious to brain, visceral and pancreatic tissue in animal models, since periodic breathing patterns are essentially intermittent hypoxia exposures; exposure to repeated intermittent hypoxia even for short periods of a few hours results in severe cerebellar injury (16), and damage to medullary and peripheral cardiovascular integrative sites (34). In addition, reduced bone development occurs (35), as well as damage to structures mediating glucose control (36). Intermittent hypoxia episodes in human neonates lead to acute and chronic morbidities, including retinopathy of prematurity, impaired growth and cardiovascular regulation, bronchopulmonary dysplasia, sleep disordered breathing and neurodevelopmental disabilities (37–41).

Interventions to maintain ventilation in the neonatal period are few, and have the potential to impose injurious consequences. Continuous positive airway pressure (CPAP) procedures in infants pose risks, especially in the long term due to the fragility of the lungs in neonates (42) as well as concurrent bone distortion from facial masks on facial structures in early development (43). The consequences of using CPAP to manage periodic breathing in adults raise other concerns, although these aspects have not been fully explored in neonates. In adults, CPAP is often ineffective to stop Cheyne-Stokes breathing in heart failure patients (44, 45). In sleep-disordered breathing patients, major cardiovascular issues arise from use of servo-controlled positive pressure ventilation in those with periodic or Cheyne-Stokes breathing; positive pressure ventilation use leads to increased mortality in those with such breathing patterns [for rationale, see (46)]. Although adult conditions may not exactly parallel those encountered in infants, the absence of data during early development should not reduce concern of consequences of such interventions.

A common intervention to support neonatal breathing is to use methylxanthines, including caffeine, aminophylline, or theophylline, to enhance breathing drives, principally through neurotransmitter arousal processes. The benefits of caffeine, especially with early use reducing bronchopulmonary dysplasia, have been described (47). However, the long-term consequences of such use remain unclear; although a large study (48), found little change of sleep characteristics or obstructive sleep apnea in later childhood following treatment of caffeine or positive pressure ventilation to premature infants. However, concomitant arousal effects of caffeine enhance the risk for diminished sleep state integrity in prematurity, with unpredictable consequences to later cognitive development (49, 50). Thus, interventions to support breathing in premature infants might consider options other than caffeine or positive pressure ventilation to address issues of maintenance of respiratory muscle tone to prevent airway collapse, stimulation to respiratory drives to avoid central apnea, as well as synchronization of control systems to prevent periodic breathing.

Several types of impaired breathing must be considered in neonates and young infants. The first relates to loss of one or more critical drives that lead to hypoventilation or central apnea, a pattern typically found with loss of chemical sensing or temperature drive, a second relates to airway obstruction from loss of drive to the upper airway muscles with continued diaphragmatic efforts, a pattern commonly found in REM sleep or with cerebellar injury (2), and a third type of periodic breathing which stems from a loss of coordination or timing of central drive on both upper airway and diaphragmatic musculature. A solution to the first two of these issues would be to substitute a missing drive by enhancing the action by another, perhaps less commonly used influence. Correction of periodic breathing can be accomplished by assisting cerebellar-pontine mechanisms in coordination actions to overcome the stop-start patterns of periodic breathing, and stabilizing the regularity of breathing patterns. There are rather simple means to implement both solutions.

**Figure 1:**
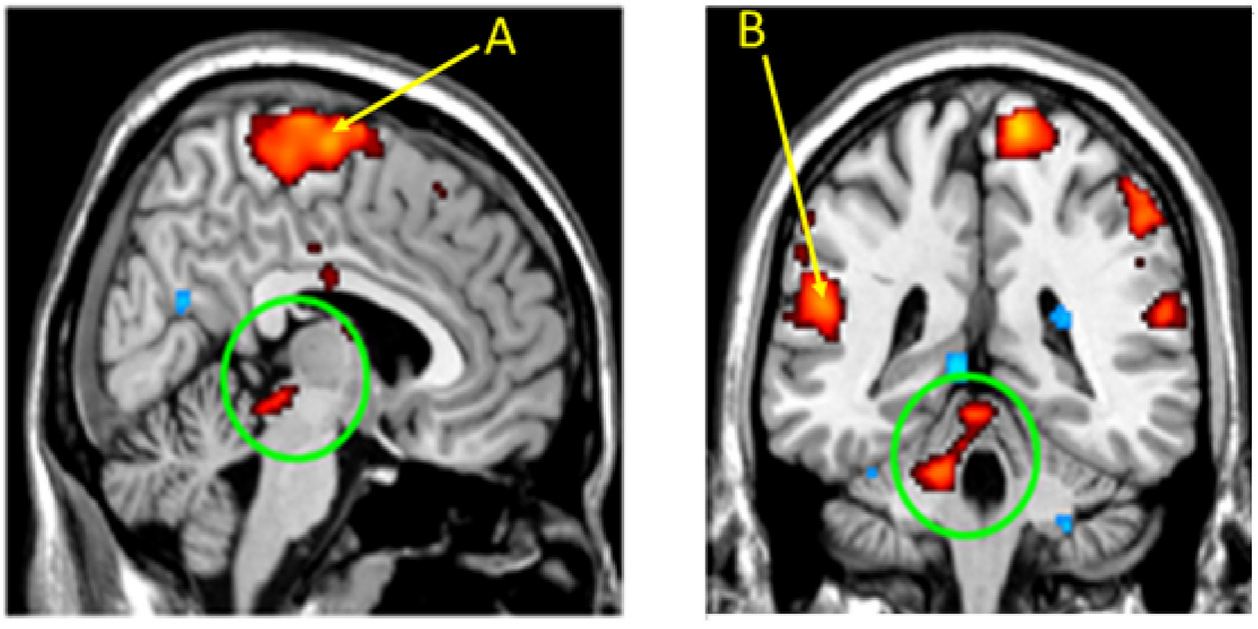
Cerebellar and parabrachial pontine nuclei are activated by limb proprioceptive sensory stimulation. Functional MRI images following passive right foot movement in 14 children activated the parabrachial pons (Left circled area) and medial cerebellum (right circled area), sites involved in respiratory timing and blood pressure control. The sensorimotor cortex for the foot **(A)** and for the cervical region (for diaphragm), **(B)** are also activated, indicating that locomotion (foot) and breathing motor sites are concurrently recruited by the proprioceptive stimuli. From (51).

The evidence for coupling of breathing and limb movement processes derives from both animal physiological studies and human functional magnetic resonance studies (fMRI). We used fMRI procedures to validate in humans that lower limb proprioceptive stimulation activates cerebellar and parabrachial pontine structures (51) (Figure 1, left and right circled areas). In addition, the cortical area representing foot movement (Figure 1A) is activated, as is the cortical cervical motor area for the diaphragm (Figure 1B) (cervical nerves 3, 4, and 5 innervate the diaphragm).

Any intervention to support breathing must consider both the drive to upper airway muscles as well as timing of drive to those muscles. The drive aspect requires the full array of influences from airflow sensors, temperature influences, lung stretch receptors and CO_2_ sensors for appropriate action, any of which can be poorly developed in infants. The timing aspects rely on appropriate development of cerebellar and pontine interactive circuitry, as well as chemical sensing from the periphery and central chemosensors, both of which can be distorted in timing during newborn development (52).

## Overcoming Lost or Reduced Respiratory Drives

A long history of experimental evidence indicates that a variety of “non-classical respiratory” sensory inputs can stabilize breathing patterns. Those interventions can take the form of detecting apnea, and then introducing an arousing stimulus, typically through vibration to recruit the “wakefulness stimulus” and thus enhance breathing (53). Cutaneous stimulation through rubbing of peripheral limbs has been successfully used (54), and whole-body vibratory stimulation has also been applied intermittently through vibratory mattresses (55), and lessens apnea. Vestibular stimuli, applied by oscillating a water bed (56), or manual rocking (57) show remarkable effectiveness in stabilizing breathing. There are obvious logistic issues with continuous manual rubbing intervention, incorporating whole-bed vibration, or rocking, but the interventional studies indicate that efforts to restore breathing do not necessarily have to depend on manipulating chemosensitive processes or altering arousal-related neurotransmitters through methylxanthines, or using potentially injurious positive pressure ventilation techniques.

One solution to providing another breathing drive is to use a phylogenetically old reflex that links limb locomotion with increased breathing muscle activity; running imposes increased ventilatory demands for metabolic reasons, and breathing must often increase immediately, e.g., to escape from a predator, with no time available to build up CO_2_ signaling to breathe faster. This reflex thus bypasses the normal, but sometimes lost or reduced chemosensing and temperature drives that can occur in neonatal life or in genetic errors, such as CCHS. CCHS provides an “experiment of Nature” that allows evaluation of other means to support breathing; the condition, a consequence of PHOX2B mutation, shows an absence of CO_2_ and O_2_ sensitivity (3) and impaired temperature regulation, among other autonomic deficits (21). The neuromodulatory aspects of breathing support for diminished chemosensitivity were partially drawn from fMRI studies of CCHS patients who showed the reduced participation of defined cerebellar and parabrachial pontine areas to CO_2_ challenges (58).

Early recognition of the limb movement/breathing relationship in CCHS sprung from studies relating body movement or passive limb movement to breathing (22, 59); CCHS patients who would turn blue if watching television, would ventilate normally if playing soccer. Even passive foot movement during sleep was effective in supporting breathing (23). The locomotion-breathing reflex recruits not only the diaphragm but also the upper airway and thoracic wall muscles, and can overcome the other-than-diaphragm respiratory muscle paralysis of REM sleep as well as alterations in temperature and chemical senses of that sleep state. The recruitment of upper airway muscles in addition to the diaphragm overcomes the possibility of causing obstructive apnea—unlike phrenic nerve stimulation which can result in upper airway collapse by generating a too-high negative pressure (60).

The intervention also assists timing of breathing by directly activating the cerebellum and parabrachial pons, brain sites that coordinate proprioceptive and other input to synchronize activity of the respiratory muscles. This timing coordination is critical for resolving periodic breathing, which typically results from a temporal mismatch of CO_2_ sensing between the carotid and central chemoreceptors. CCHS patients show profound injury in cerebellar sites (21, 58, 61, 62), likely a consequence of the PHOX-2B relationships to that structure and unintended failure of ventilory support, triggering hypoxic episodes. The demonstration that foot movement can recruit enhanced breathing efforts is useful to show the principle of the phylogenetically old reflex, but that physical process of movement is obviously impractical for neonatal use. However, a variety of electrical or mechanical means can be used to simulate foot or hand movement, i.e., activate proprioceptive fibers to “trick” the brain into triggering the limb movement-breathing coupling reflex. Vibration of the foot or hand will activate proprioceptive fibers carried to the pontine and cerebellar nuclei, simultaneously recruiting action in breathing nuclei of the medulla and pons, and thus activating upper airway muscles, thoracic wall muscles, and the phrenic motor pool. These processes can thus provide a “drive” to those breathing muscles, and by activating upper airway muscles, overcome the potential for obstructive sleep apnea. In addition, the diaphragmatic and thoracic wall musculature which fail in central apnea can be excited (63, 64). The neuromodulatory procedure may have particular value in other conditions, such as drug-resistant epilepsy, where a concern exists for sudden unexpected death in epilepsy (SUDEP), an outcome typically dependent on the frequency of seizures, with such seizures often accompanied by hypoxic or hypotensive periods (65).

## Intervention Device

**Figure 2:**
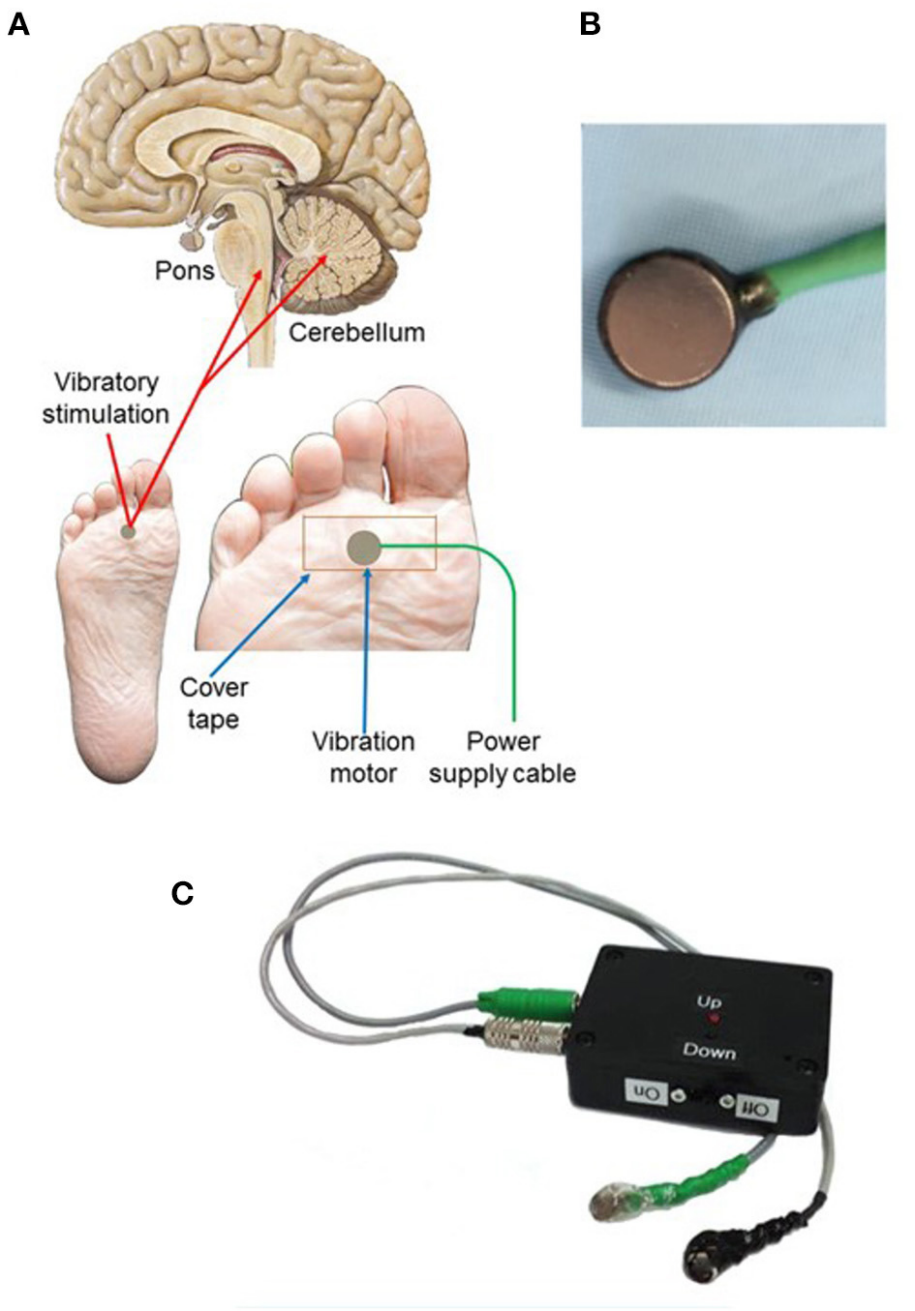
**(A)** Placement of vibrator motor on the sole of the foot; since the limb movement/breathing reflex is operative on all four limbs (from early origins of the reflexive coupling), placement on the palm of the hand is also effective. The activated proprioceptive fibers project to the cerebellum and parabrachial pons, that in turn, integrate with motor action to the upper airway and diaphragm. **(B)** Vibrator motor with power lead. **(C)** Power supply containing two 1.5-volt fused AA batteries with two levels of vibratory stimulation. From (65).

Recruitment of proprioceptive fibers to activate breathing requires only simple vibration to mechanical receptors of the limbs. Such a device is shown in Figure 2. The objective is to apply vibration to the sole of the foot (or palm of the hand) to activate proprioceptive nerve signals, normally activated by walking or running, that rise through spinal pathways to the pons and cerebellum (Figure 2A). The cerebellar and parabrachial pontine sites coordinate activation of oropharyngeal muscles, including the genioglossal fibers of the tongue and muscles of the diaphragm. The vibratory device consists of small vibratory motors (Figure 2B) that are taped to the sole of the foot by tape. The vibratory motors, attached to the leads, are powered by two alkaline batteries, fused for safety, from a power supply box (Figure 2C), providing 1.5 or 3.0 volts power (switchable). The motors provide two levels of vibration amplitude at 128 Hz, a standard vibratory signal used to elicit reflexes in neurological testing. The waveform and frequency characteristics were determined after extensive empirical trials, with attention to tolerance of vibratory levels (66).

## Periodic Breathing

By providing excitation to the cerebellum and parabrachial pons, timing and synchronizing effects are enhanced through proprioceptive stimulation of the limbs, and thus can reduce or abolish periodic breathing, a major concern in premature neonates, since such respiratory patterns result in serious intermittent hypoxia. The intervention can also improve cardiovascular aspects accompanying apnea, reducing bradycardia associated with the stopped-breathing periods, and assisting perfusion.

**Figure 3:**
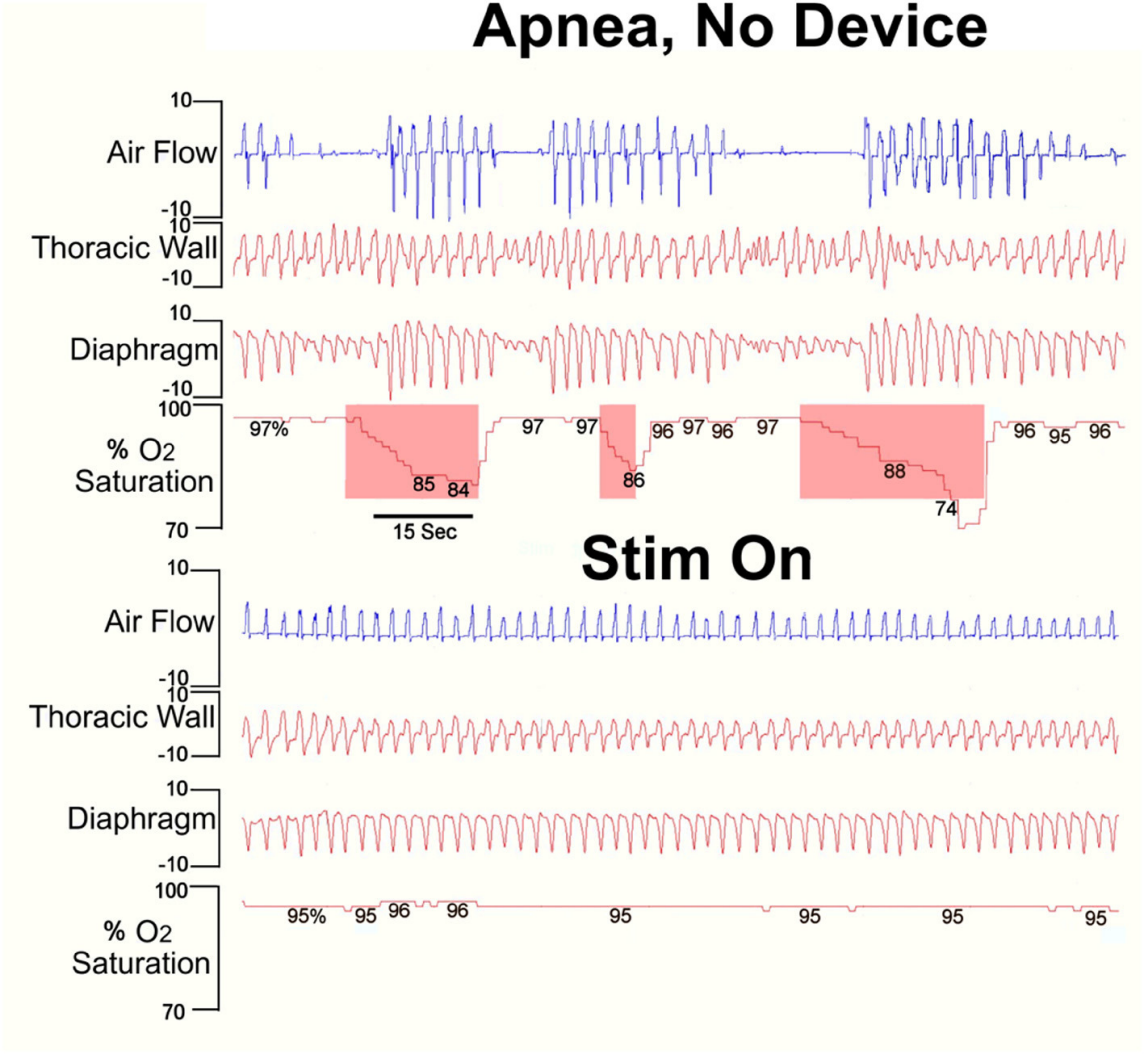
Stimulation of proprioceptive fibers of the foot of a pediatric (4 years) CCHS patient without (top) and with the device (Stim On). Note the decline in O_2_ saturation (shaded O_2_ saturation areas following cessation of breathing efforts) during no stimulation, and the complete abolition of periodic breathing and restored saturation with stimulation. Clinical data courtesy of Dr. M. Woo [Derived from (66)].

The usefulness of the intervention in managing periodic breathing can be seen in Figure 3, which shows breathing in an awake CCHS child with and without the vibratory device. The CCHS patient desaturates to low 70% values, but those values remain at full saturation with stimulation.

The device applies transcutaneous vibration to the soles of the foot and palms of the hand to elicit nerve signaling from pressure and other limb proprioceptor sensors to pontine, cerebellar, and medullary brain areas that coordinate limb movement and reflexively activate brain areas controlling breathing. The procedure enhances a reflexive drive to breathing when other breathing drives fail from disease processes or during sleep. The device is of use in obstructive sleep apnea, central apnea, periodic breathing, and hypoventilation, and will also normalize extremes of change in blood pressure to respiratory events (63, 64, 66).

It is important to note that the mode of action used by recruitment of reflexive breathing drives differs from the intuitive mechanism of arousing the apneic subject to restore the “wakefulness” drive to breathe. Such an approach can be effective for restarting breathing, but repeatedly arouses the subject, making a night's sleep not restful. Instead, the principle is to recruit an ancient reflexive drive that maintains sleep integrity (24, 63).

**Figure 4:**
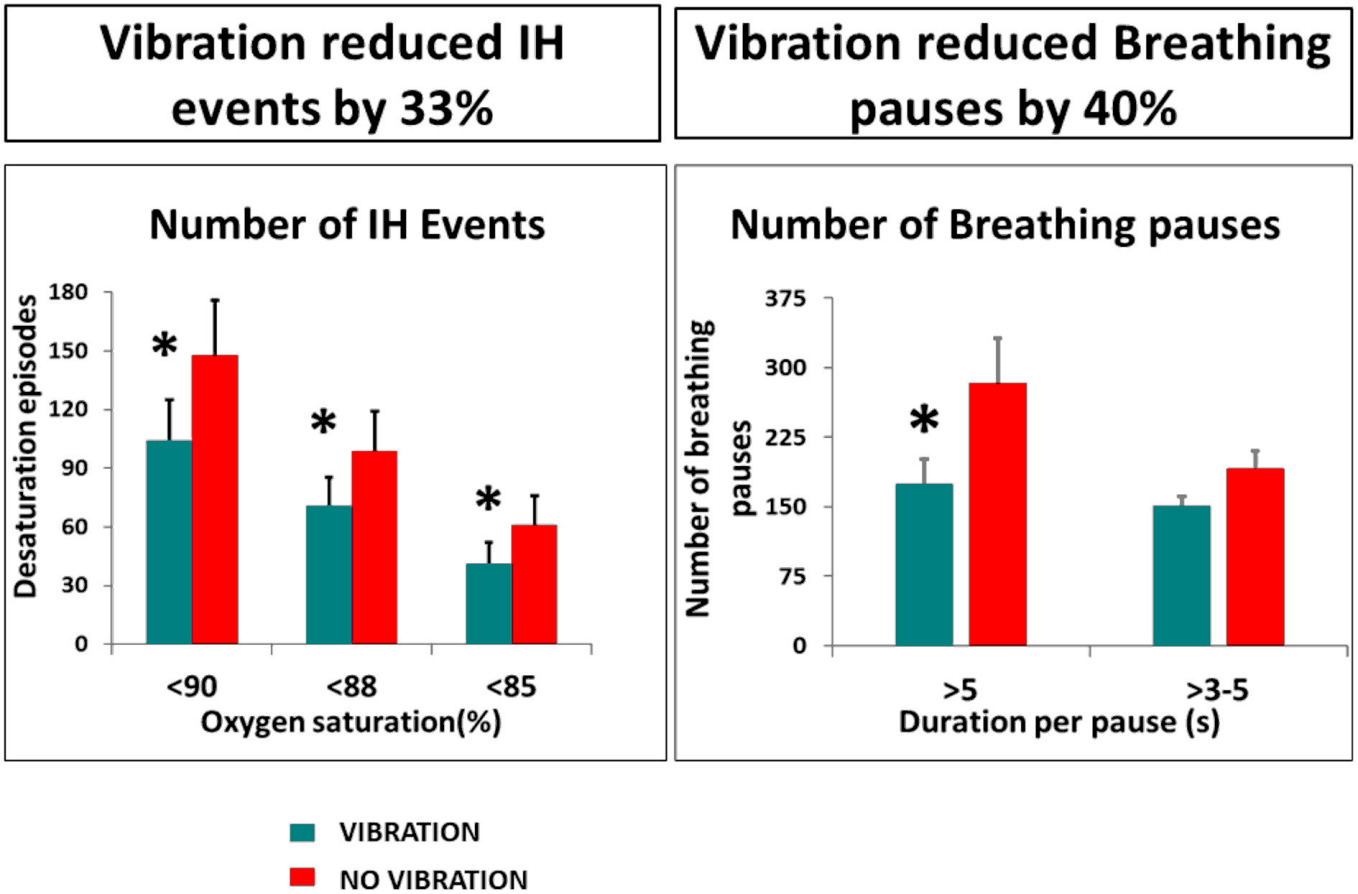
Vibratory proprioceptive stimulation in 15 infants with apnea of prematurity reduced intermittent hypoxic events (IH) by 33% and breathing pauses by 40%. *p <0.05. From (63).

**Figure 5:**
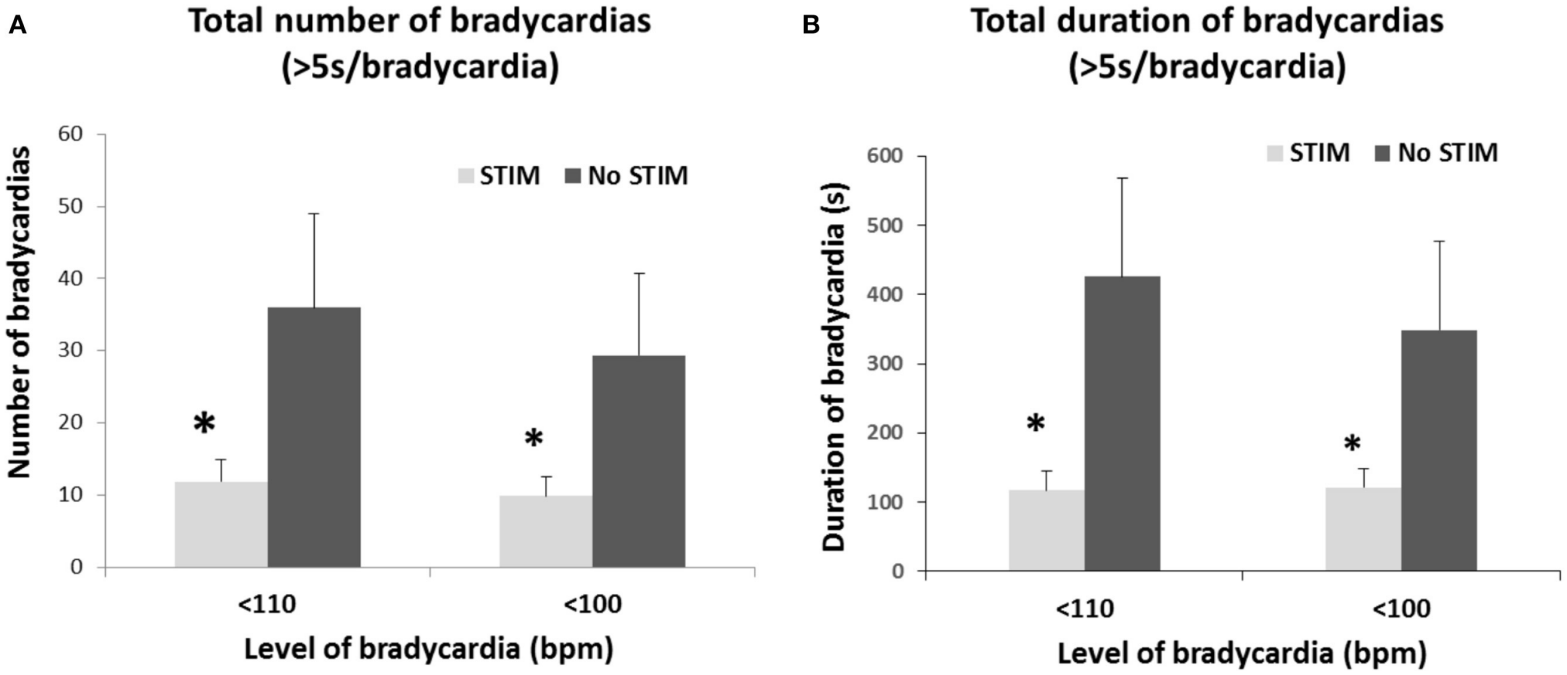
Limb proprioceptive stimulation in premature infants reduced the total number **(A)** and the total duration **(B)** of mild (<110 bpm) and moderate (<100 bpm) bradycardic episodes from pre-stimulation periods. A 3-fold reduction in total duration of both mild and moderate bradycardia episodes occurred. *p < 0.05. From (63).

The safety, suitability and efficacy of the procedure has been shown in a premature infant trial (63), that demonstrates a significant reduction in number and duration of long breathing pauses and intermittent hypoxic events, as well as the number and duration of bradycardic events (Figures 4, 5). The intervention provides an ancillary drive to breathing which replaces missing drives during development, allowing stabilization of breathing, diminishing apnea, and reducing extreme changes in cardiovascular patterns accompanying breathing pauses.

## Alternative Options for Neonatal and Adult Apnea

The concerns for breathing support in neonates also extend to adults with sleep-disordered breathing. The principal intervention has been CPAP, which has serious deficiencies in patient adoption, since the devices are uncomfortable, are often noisy, are not compact, making travel difficult, has humidity control issues, and long-term support for blood pressure is not assured (67, 68), and can be dangerous for periodic breathing use (46). Mandibular positioning devices have been adopted for mild or moderate adult OSA, but are not considered for neonatal cases, and are often considered inadequate for severe adult apnea. Such devices pose a potential for temporal mandibular joint injury (from forward positioning of the mandible), and do nothing for central apnea (in conditions where patients lack central drive to breathe for all respiratory muscles, not just upper airway musculature). Genioglossal/hypoglossal nerve stimulation requires invasive surgical implantation of an electrical stimulation device, coupled with placing leads to the 12th cranial nerve or to genioglossal fibers of the tongue. The procedure is inappropriate for neonates. The surgery is expensive, is coupled with a risk for infection, damage to nerves and other tissue from stimulation leads, and may require multiple surgical interventions to restore the subcutaneous power supply of the implanted device. Mechanical positive pressure ventilation can be used, but also poses a concern of injury to delicate or compromised lungs, such as those in young or medically-compromised patients.

## Conclusions

Disordered breathing in early life can lead to impaired oxygenation, often with intermittent hypoxia exposure, which can induce severe injury to multiple brain sites. Disordered breathing can result from failure of chemical, temperature, or state-related motoric drives; particular sleep states enhance the appearance of some of these failures. Alterations in drives can further compromise breathing by delayed timing or inappropriate coordination of afferent influences, or disrupt integration of sensory input with motor outflow to upper airway and diaphragmatic muscles. Multiple interventions have been used to demonstrate how potential injury from positive pressure procedures or bypassing caffeine administration can be avoided. These interventions attempt to modulate sensory input, include tactile and vibratory bed stimulation, and often rely on recruiting arousal actions to restore breathing. Vestibular input, applied through oscillatory water beds or rocking, has been employed successfully to exert excitatory vestibular/cerebellar processes to activate and synchronize breathing with motion input. Loss of breathing drives can be overcome by enhancing ancient reflexive interactions between limb locomotion and respiration, and that enhancement can be accomplished artificially by proprioceptive activation, “tricking” the brain into perceiving that the limbs are moving. If sufficient stimulation is provided, the proprioceptive signals can enhance cerebellar and pontine processing to improve coordination of integrative processes and abolish periodic breathing.

## Author Contributions

RH and KK contributed equally to the design and implementation of the research and findings outlined in this manuscript. Both authors contributed to the article and approved the submitted version.

## Funding

This research was supported by grants from the UCLA Children's Discovery and Innovation Institute's Seed Grant award, Today's and Tomorrow's Children's Fund Award, 2016 and Little Giraffe Foundation NICU Support Grant, 2016 to KK and by the Fidelity Charitable Nancy Adams and Scott Schoen Fund and the Kraig and Linda Kupiec Family Trust to RH.

## Conflict of Interest

The University of California has been issued a patent for a device described in this review, listing RH as one of the inventors. The remaining author declares that the research was conducted in the absence of any commercial or financial relationships that could be construed as a potential conflict of interest.

## Publisher's Note

All claims expressed in this article are solely those of the authors and do not necessarily represent those of their affiliated organizations, or those of the publisher, the editors and the reviewers. Any product that may be evaluated in this article, or claim that may be made by its manufacturer, is not guaranteed or endorsed by the publisher.
